# Systematic evaluation of common natural language processing techniques to codify clinical notes

**DOI:** 10.1371/journal.pone.0298892

**Published:** 2024-03-07

**Authors:** Nazgol Tavabi, Mallika Singh, James Pruneski, Ata M. Kiapour

**Affiliations:** 1 Boston Children’s Hospital, Boston, MA, United States of America; 2 Harvard Medical School, Boston, MA, United States of America; West Pomeranian University of Technology, POLAND

## Abstract

Proper codification of medical diagnoses and procedures is essential for optimized health care management, quality improvement, research, and reimbursement tasks within large healthcare systems. Assignment of diagnostic or procedure codes is a tedious manual process, often prone to human error. Natural Language Processing (NLP) has been suggested to facilitate this manual codification process. Yet, little is known on best practices to utilize NLP for such applications. With Large Language Models (LLMs) becoming more ubiquitous in daily life, it is critical to remember, not every task requires that level of resource and effort. Here we comprehensively assessed the performance of common NLP techniques to predict current procedural terminology (CPT) from operative notes. CPT codes are commonly used to track surgical procedures and interventions and are the primary means for reimbursement. Our analysis of 100 most common musculoskeletal CPT codes suggest that traditional approaches can outperform more resource intensive approaches like BERT significantly (P-value = 4.4e-17) with average AUROC of 0.96 and accuracy of 0.97, in addition to providing interpretability which can be very helpful and even crucial in the clinical domain. We also proposed a complexity measure to quantify the complexity of a classification task and how this measure could influence the effect of dataset size on model’s performance. Finally, we provide preliminary evidence that NLP can help minimize the codification error, including mislabeling due to human error.

## Introduction

Advancements in natural language processing (NLP) have led to increased interest in health care research and quality improvement. The development of automated pipelines to process large volumes of clinical notes can optimize healthcare operation (e.g., quality improvement, resource management, billing) and improve clinical care (e.g., evidenced-based clinical decision making). As up to 80% of the electronic medical record is comprised of this unstructured documentation [1, 2], NLP is postured to become an increasingly valuable tool for processing this data.

Codification of diagnoses (ICD, International Classification of Diseases) or procedures (CPT, Current Procedural Terminology) from clinical and procedure notes offer a unique opportunity to systematically assess the performance of NLP techniques in clinical domains. Efficient and accurate coding of clinical notes (e.g., billing or diagnostic codes) impacts the entire healthcare industry, including healthcare systems, care givers, insurance companies, and policy makers. The practice of assigning codes is a complex and labor-intensive process that is prone to human error. Failure to code correctly can result in inadequate patient care and may lead to increase in expenses or delays in the reimbursement process. Recent studies regarding the automation of the coding process have tried an array of techniques, ranging from traditional text matching to deep learning-based approaches, to categorize clinical notes [3–11]. [3, 4] provide literature reviews of NLP techniques applied on clinical notes. While [6–9, 11] propose new approaches to codify them and [12, 13] use SNOMED-CT (Systematized Nomenclature of Medicine Clinical Terms) and UMLS (Unified Medical Language System) to identify key-terms in clinical notes. Yet, there is a paucity of studies to systematically evaluate their relative performance in clinical domain.

The purpose of the current study is to systematically evaluate the ability of commonly used NLP techniques to predict CPT codes from unstructured operative notes. Operative notes are a specific subgroup of clinical notes that contain details of surgical procedures performed on patients and the primary source for CPT billing code assignment. The clinical importance of operative notes along with their one-to-one relationship with CPT codes offer a unique opportunity to evaluate NLP performance in clinical domain. In this study, we compared the performance of three common NLP techniques (Term Frequency-Inverse Document Frequency (TF-IDF) [14], Doc2Vec [15], and Bidirectional Encoder Representations from Transformers (BERT) [16] to predict the 100 most common musculoskeletal CPT codes in a high-volume multi-site academic pediatric and young adult orthopedic surgery and sports medicine clinic. These approaches were chosen because they are frequently used on clinical data [3, 10, 17] and each represent different eras of advances in NLP. TF-IDF is a traditional NLP approach, however with recent advancements, it is still tough to beat in some applications. DOC2Vec is one of the early NLP approaches with neural networks. It was introduced after the very celebrated Word2vec [18] approach and is still one of the few methods that provide sentence-level embeddings, whereas other methods provide word-level embeddings. BERT [16] is, one of the the most used models for NLP tasks [19], and earliest and smallest in the family of LLMs. Because of its architecture and number of parameters, although it requires more resources than previous methods, it is far less resource intensive compared to more recent LLMs such as chatGPT [20] and Llama 2 [21].

**Fig 1:**
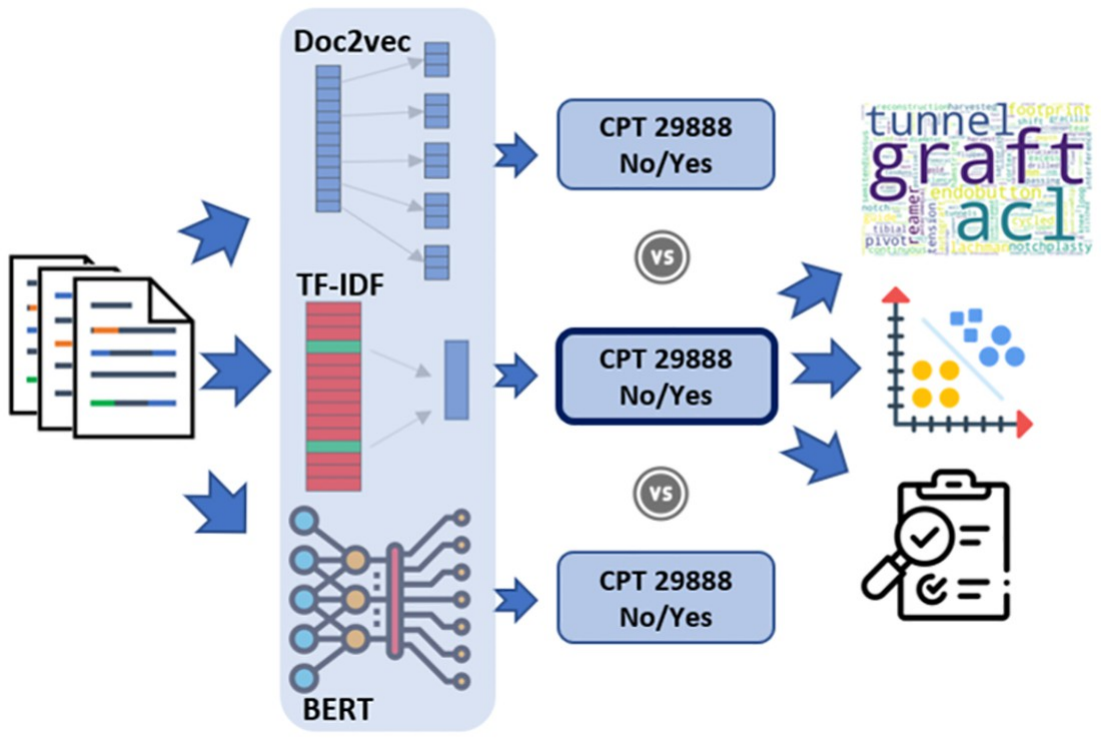
CPT predictions from clinical notes were tested using 3 different approaches, TF-IDF, Doc2vec, and BERT. These experiments were performed on 100 most common CPTs in musculoskeletal operative notes. In this figure it is shown for CPT 29888 as an example. Afterwards, the best performing approach, TF-IDF with feature selection, was analyzed for interpretability, CPT complexity, and possibility of being used for quality control of CPT assignments.

We further studied text-related factors influencing the model performance (i.e., complexity of CPTs). We hypothesized that NLP models could predict CPT codes with near-human accuracy, and that the state-of-the-art embedding-based models (e.g., BERT) would outperform traditional NLP approaches (e.g., TF-IDF). Secondarily, we hypothesized that increased CPT complexity would be associated with decreased model performance. Diagram in Fig 1 shows a summary of the study design.

## Methods

### Data

Following IRB approval (IRB-P00037878), operative notes from patients with at least one encounter at any of the six Boston’s Children Hospital Orthopedic Surgery and Sports Medicine clinics from 2010—2020 were acquired (n = 126,789 documents). The operative notes were filtered to include only procedures on the musculoskeletal system (CPT codes 20100—29999), resulting in 44,002 notes. The 100 most prevalent CPT codes in our dataset (S1 Table) were used as labels for the classification tasks.

### TF-IDF

TF-IDF is a statistical method for extracting features, or keywords, from text [14]. With this method, documents are represented as vectors where each feature (dimension) in the vector space corresponds to a unique term in the dataset, meaning the number of features is equal to the number of terms in the dataset, which is often quite large and can lead to overfitting and slow learning [22]. To combat this, we applied feature selection on TF-IDF vectors with different configurations. The classifications were done on the following four feature sets:

1. All extracted features.
2. Top 100 most relevant keywords to the target CPT.
3. Top 500 most relevant keywords to the target CPT.
4. Top K most relevant keywords, where K is a hyper-parameter optimized for each CPT to get the best performance.

Most relevant keywords were identified based on F-values from ANOVA test. In setting 4, the search space for K was set as log-uniform so that the model has a higher chance of assigning a lower number to K to avoid overfitting.

### Doc2vec

Doc2vec [15] is a generalization of Word2vec [18], which is a technique used to represent words as vector embeddings (points in a high-dimensional space) such that similar words have representations that are closer to each other. Doc2Vec uses the word embeddings generated by word2vec to vectorize the entire documents. Word2vec and Doc2vec use shallow networks to learn the context of words and sentences. To train Doc2vec representations, the following hyper-parameters were set:

- D: dimension of the embeddings
- W: window size (the W surrounding words which is referred to as context in the description of Doc2vec.)
- DM versus DBOW: Optimization approach, using either Distributed Memory or Distributed Bag of Words

### BERT

BERT is an NLP model which has been extensively used in multiple text processing domains and NLP tasks [16]. It is a deep neural network with multiple layers of encoders with bidirectional self-attention heads. Because of its many layers, BERT models require a large amount of training data. Hence, researchers have pre-trained the BERT models on more specific datasets, as to improve the model’s performance for specific tasks. In this study we use Clinical-BERT [23], which is pre-trained on publicly available MIMIC clinical notes [24]. It’s worth noting that while BERT models can process sequences with max length of 512 tokens, clinical notes are usually longer. The most common solution to this problem is to truncate the note, however since the details of the surgical procedure, required for proper prediction of the CPT code, are spread across the note this might result in loss of valuable information. Aside from that, training an end-to-end process for these tasks (fine-tuning the BERT model) does not give acceptable results, because of highly imbalanced data. The most common CPT in this study (20680), has a ratio of 10% to 90% and the least common CPT (25390), has a ratio of 0.2% to 99.8%. Even by changing the loss to weighted cross-entropy, the end-to-end BERT classification is not able to converge for most of the CPTs.

To take advantage of BERT embeddings and make sure all the information in the text is captured, notes were broken down to sub-sequences of less than 512 tokens, and their embeddings were extracted and given to a classifier.

For getting note level embeddings based on word (token) level embeddings, several approaches have been previously suggested. Including averaging the embeddings of all words in the document, taking the maximum word embedding in the document as a way to select the most important features [25], or using the embeddings of a special token called the [CLS] token as a general representation of input sentence [26, 27]. Here we generated the note representations using all of these approaches and compared their performance in predicting the CPT codes. The following notation was used:

- A_W: Average token/word embeddings
- M_W: Max of token/word embeddings
- A_CLS: Average of [CLS] embeddings
- M_CLS: Max of [CLS] embeddings
- X + Y: Concatenate embeddings of X and Y,
  (X, Y ∈ {A_W, M_W, A_CLS, M_CLS})

### Classification

For experiments in this study Support vector machine (SVM) with RBF kernel [28] was used as the classifier. The features from one of the aforementioned approaches (i.e., TF-IDF, Doc2vec, and BERT) were used as input to the classifier to predict the CPT codes. Separate classifiers were trained for each CPT. Using the same classifier for all approaches enabled us to isolate the effect of the feature extraction approach on model performance. Model hyper-parameters (*C* & *γ* and *K* for TF-IDF with feature selection) were optimized with Hyperopt [29], an approach for parameter tuning based on Bayesian optimization. 20% of the data (by preserving the percentage of samples for each class) was used as test set and the training data was split into 5-fold cross-validation for tuning the hyper-parameters. After choosing the best hyper-parameters, the model was trained on the train set and evaluated on test set.

### Model performance evaluation

We assessed the model’s performance in predicting the CPT codes with the area under the receiver operating characteristic curve (AUROC). We also calculated the accuracy, sensitivity, and specificity across all predictions. We first compared the performance within each feature extraction approach (i.e., TF-IDF, Doc2vec, and BERT) and then compared the best-performing models from each approach together. These comparisons were made using Critical Difference Diagram [30, 31]. The Critical Difference Diagram is a method to compare multiple classification approaches over different tasks, and it ranks them based on AUROC (rank 1 indicating the best performing approach) from right(best) to left(worst) and denotes a lack of significant difference in AUROC by connecting the similar approaches with a thick horizontal line. Critical Difference Diagram is computed based on the Friedman test and a post-hoc analysis based on the Wilcoxon-Holm method. All P-values are two-sided and significant at P<0.05.

### CPT complexity and procedure prevalence assessment

In order to describe the performance variance over different CPTs, we introduced the CPT complexity measure. Each NLP approach generates a feature space in which clinical notes are points in. Neighbors of a point are points (notes) with lowest Euclidean distance to it in that feature space. The proposed complexity measure is composed of two metrics defined on this feature space. Consider a single note (text). A CPT is considered simple (not complex) when 1- the note’s neighbors have the same label (CPT). 2-The notes with the same label (CPT) are closer to each other in terms of distance. The same concept is described in more mathematical terms below.

For each given CPT, notes are either positive or negative, meaning they are assigned that CPT or not accordingly. Measures were first calculated for each positive note and then averaged.

- *Average same label neighbor ratio*: For each positive note, *M* nearest neighbors were identified, *M* being 10% of overall positive samples for each CPT. (e.g., for a CPT with 4470 positive cases, 447 nearest neighbors were considered). The ratio of the same label neighbors within the 10% neighbors was then calculated ($\frac{\#\text{positive}\;\text{neighbors}}{\#\text{neighbors}}$).
- *Average same label neighbor distance*: For each positive note a sphere was established (with that particular note as the center) to contain 10% of the nearest same label neighbors. The radius of the sphere was then normalized by the longest distance between two notes in the dataset.

A higher *average same label neighbor ratio* and a lower *average same label neighbor distance* indicate that the positive samples are better clustered together in the space (more condensed and separated clusters), which in turn makes it easier to distinguish them from negative ones. To get a single measure for computing complexity we combined the two measures ($\text{log}(\frac{\text{average}\;\text{same}\;\text{label}\;\text{neighbor}\;\text{distance}}{\text{Average}\;\text{same}\;\text{label}\;\text{neighbor}\;\text{ratio}})$).

This measure was used to categorize CPTs (i.e., low complexity, medium complexity, and high complexity), to better investigate the effect of dataset size on model’s performance.

## Results

### Model comparison/ performance evaluation

#### TF-IDF

**Fig 2:**
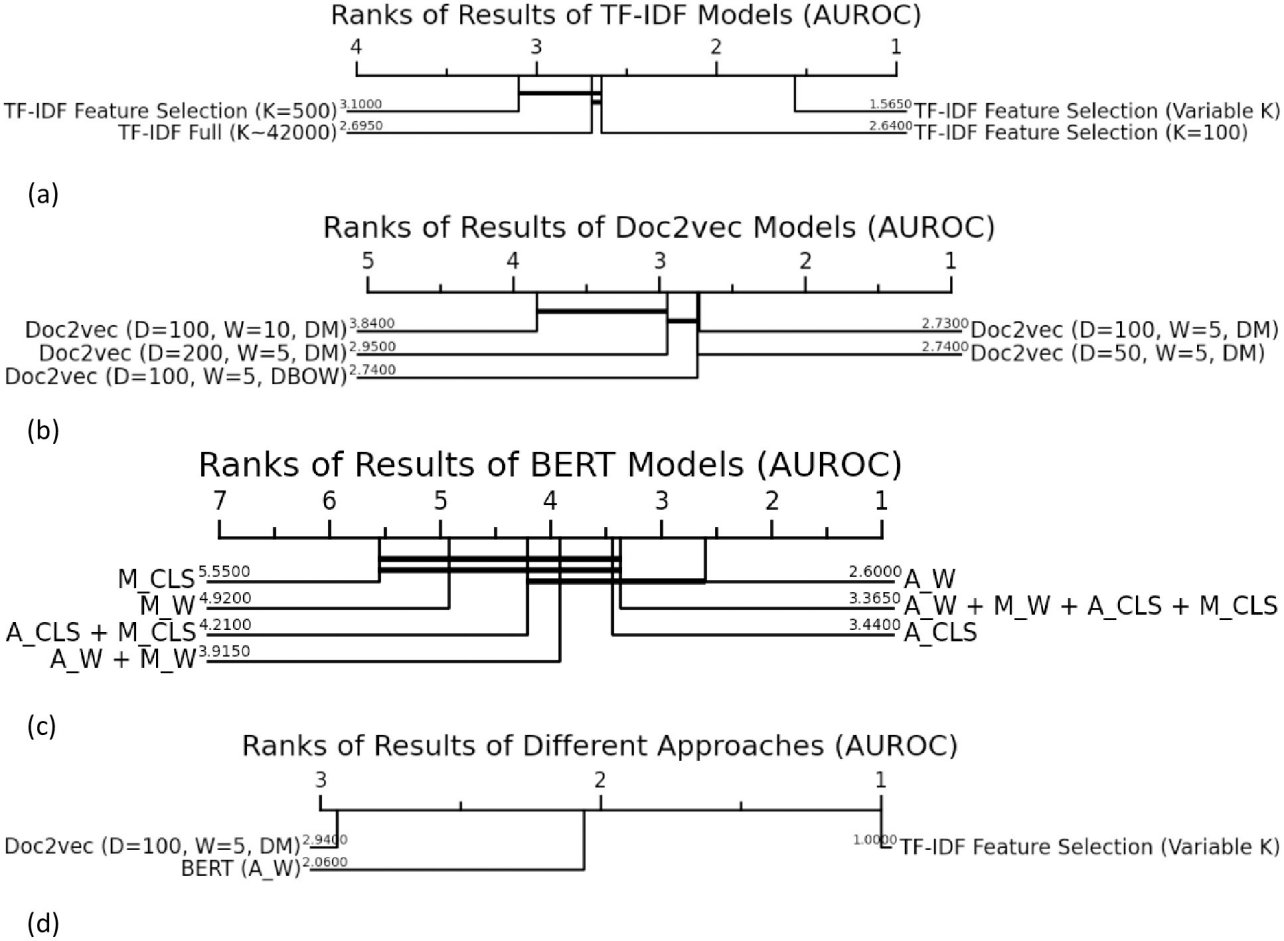
Ranking different approaches in predicting CPT codes (based on AUROC). In each diagram the approaches are ranked (rank 1 indicating the best performing approach) from right(best) to left(worst). The thick horizontal line denotes a lack of significant difference between connected groups. Plot d compares the best models from a, b, and c, with each other.

The AUROC for settings 1, 2, and 3, were not statistically significant (P>0.05). Setting 4 (classified with the K most relevant keywords) showed the best performance, with higher AUROC compared to all other 3 settings (P<3.5e-08) in Fig 2a. S2 Fig shows the relevant keywords identified for the 4 most and 4 least common CPTs (among 100) based on F-values of the ANOVA test, with larger font representing a greater weight of their F-values (relevance).

#### Doc2vec

The comparisons in AUROC between different doc2vec settings are shown in Fig 2b. As shown in the Critical Difference Diagram, changing the doc2vec parameters did not result in significant changes in model AUROC (the top 3 Doc2vec settings P>0.9).

#### BERT

Comparisons of AUROC values for the 5 tested BERT configurations are presented in Fig 2c. As shown in the Critical Difference Diagram, there were no differences in AUROC between different BERT configurations (the top 3 BERT settings P>0.6).

### Relative model performance

**Fig 3:**
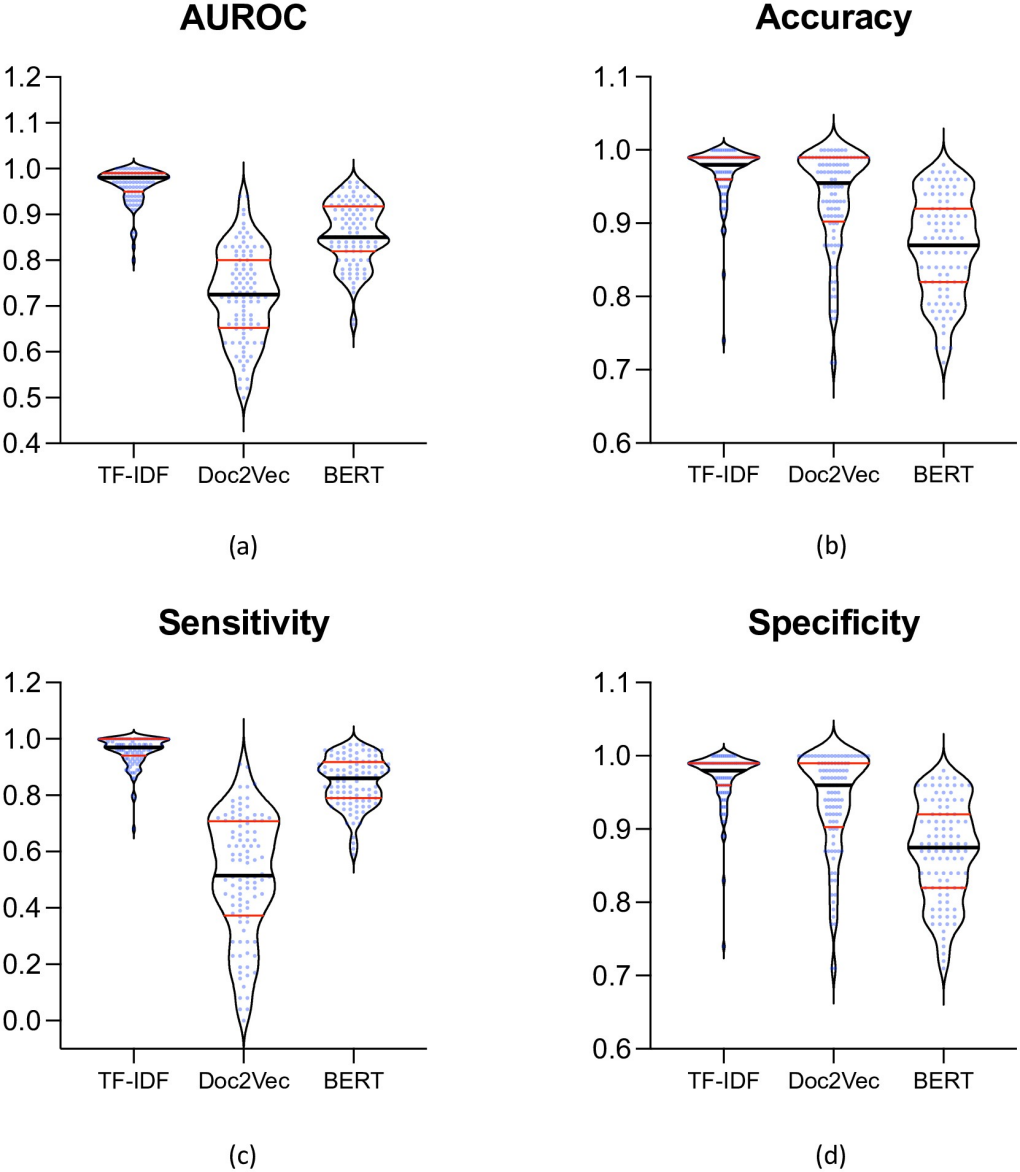
Violin plots for comparative performance metrics across three feature extraction models (TF-IDF, Doc2Vec, and BERT). The black line indicates the median and red lines indicate the quartiles.

Results on the 6 most and least common CPT codes (among 100) for the top rank method from each approach (TF-IDF Feature Selection (Variable K), Doc2Vec(D = 100, W = 5, DM), and BERT A_W) are shown in Table 1. The full results (on all 100 CPTs) are reported in the S1 Table. The statistical ranking of the best-performing models compared to each other is presented in Fig 2d. The TF-IDF approach had a significantly higher AUROC compared to the Doc2vec (by 0.24 ±0.10; P = 1.1e-17) and BERT (by 0.11 ±0.05; P = 4.4e-17). Similar trends were observed in accuracy (P<7.0e-08), Sensitivity (P<1.04e-15), and Specificity (P<5.6e-06), as seen in Fig 3.

**Table 1:** Evaluation metrics of the 6 most (top 6 rows) and least (bottom 6 rows) common CPT codes (among 100) across the classification models with TF IDF consistently performing better.

*CPT*	*Count*	*BERT*				*Doc2Vec*				*TF_IDF*			
		Acc	ROC	Spec	Sen	Acc	ROC	Spec	Sen	Acc	ROC	Spec	Sen
20680	4470	0.77	0.77	0.77	0.77	0.82	0.77	0.83	0.72	**0.97**	**0.96**	**0.97**	**0.96**
29888	3727	0.9	0.93	0.89	0.96	0.98	0.94	0.98	0.9	**1.0**	**0.99**	**1.0**	**0.99**
29881	2032	0.78	0.83	0.77	0.88	0.91	0.72	**0.93**	0.51	**0.92**	**0.95**	0.92	**0.98**
29882	1985	0.79	0.85	0.78	0.92	0.92	0.73	0.94	0.51	**0.98**	**0.97**	**0.98**	**0.96**
24538	1656	0.97	0.96	0.97	0.95	0.93	0.86	0.94	0.79	**0.99**	**0.99**	**0.99**	**0.99**
29873	1492	0.89	0.88	0.89	0.86	0.95	0.79	0.96	0.62	**0.98**	**0.97**	**0.98**	**0.97**
29065	136	0.79	0.74	0.79	0.70	**0.93**	0.67	**0.93**	0.41	**0.93**	**0.91**	**0.93**	**0.89**
27690	133	0.86	0.85	0.86	0.85	**0.99**	0.79	**0.99**	0.59	**0.99**	**0.98**	**0.99**	**0.96**
29450	131	0.87	0.76	0.88	0.65	**0.99**	0.54	**1.0**	0.08	0.95	**0.98**	0.95	**1**
25607	130	0.78	0.76	0.78	0.73	0.77	0.67	0.77	0.58	**0.98**	**0.93**	**0.98**	**0.88**
27686	128	0.87	0.91	0.87	**0.96**	**0.99**	0.59	**0.99**	0.19	0.97	**0.97**	0.97	**0.96**
25390	127	0.86	0.89	0.86	0.92	0.95	0.83	0.95	0.72	**0.99**	**0.99**	**0.99**	**1.0**

### Procedure prevalence and CPT complexity

**Fig 4:**
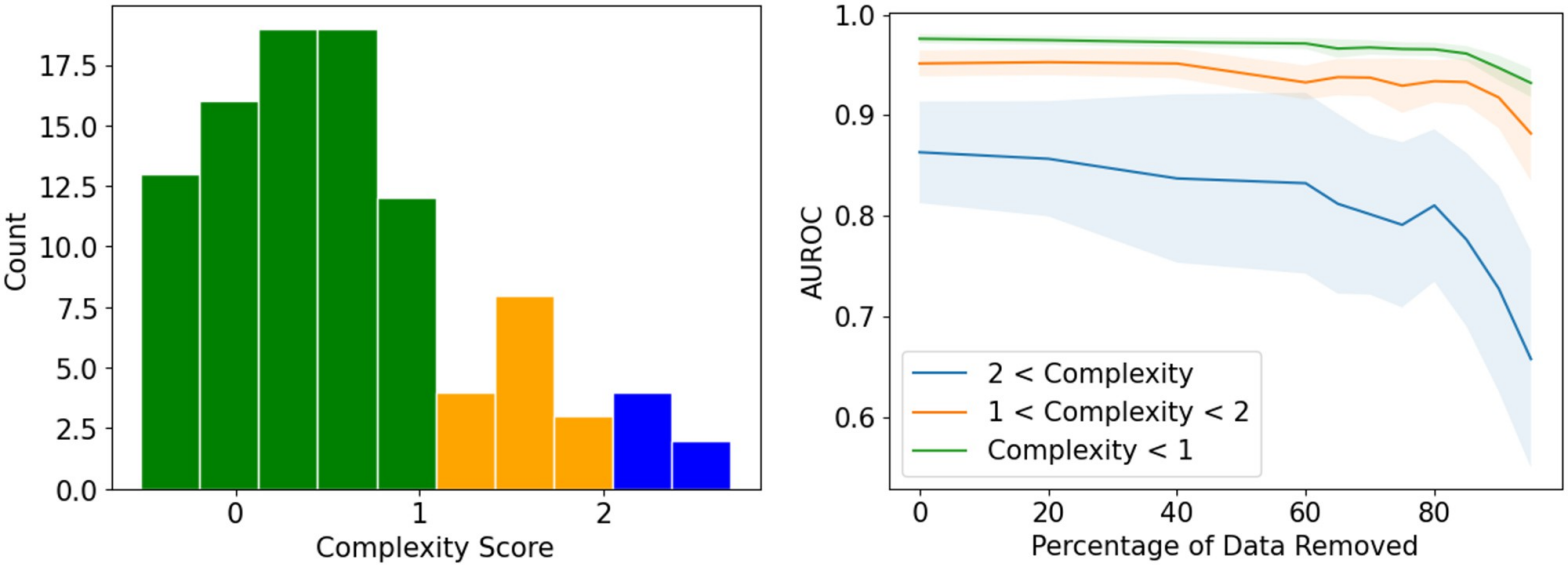
CPT complexity assessment: (a) Histogram of Complexity Scores for our 100 Most Prevalent CPT Codes. (b) Effect of the number of complex notes used during training.

The calculated complexity metrics for CPTs are presented in the S1 Table for the best performing model and S1 Fig shows a visualization of the complexity measure for two different CPTs. Table 2 shows 5 least and most complex CPTs and their descriptions, and Fig 4 shows the histogram of complexity scores for different CPT codes. As shown in Table 2, most of the CPTs with high complexity do not have a precise definition and are ambiguous. The complexity score can describe the variations between AUROC for different CPTs with adjusted R-squared of 0.611 (P < 0.001).

**Table 2:** CPT Complexity: This table lists the top 5 least and most common CPTs along with their descriptions.

*CPT*	*Complexity*	*CPT Description*
20615	-0.51	Aspiration and injection for treatment of bone cyst
26055	-0.47	Tendon sheath incision (eg, for trigger finger)
29914	-0.42	Arthroscopy, hip, surgical; with femoroplasty (ie, treatment of cam lesion)
25111	-0.36	Excision of ganglion, wrist (dorsal or volar); primary
21320	-0.35	Closed treatment of nasal bone fracture; with stabilization
20900	2.16	Bone graft, any donor area; minor or small (eg, dowel or button)
29065	2.28	Application, cast; shoulder to hand (long arm)
29999	2.33	Unlisted procedure, arthroscopy
27599	2.45	Unlisted procedure, femur or knee
27899	2.69	Unlisted procedure, leg or ankle

In this study, we look at CPTs with frequencies ranging from 127 (CPT 25390) to 4470 (CPT 20680). Considering the high degrees of variability in CPT frequencies, a question arises is that how classification results change by changing the amount of training data and whether this relationship is affected by CPT complexity. To assess the effect of procedure prevalence (count) on the model’s ability to predict the CPT, we randomly removed the notes for each procedure (CPT) by up to 90% with 10% increments and then used the reduced data to train the model and predict the CPT codes. This experiment was done with the best-performing model. The CPT codes were grouped to low complexity (complexity score<2), medium complexity (1 ≤ Complexity Score ≤ 2) and complex (Complexity score>2). The changes in AUROC relative to random reduction in data are presented (Fig 4). For low to medium complexity CPTs, the AUROC dropped only after extreme (>80%) reductions in the number of notes. However, for complex CPTs, the was a continuous decline in AUROC by reductions in number of notes with sharpest decline after 80% reduction.

### Quality assessment

In addition to being used for automating CPT code assignment, the approaches in this study can be used for quality assessment. As an example, we looked at CPT 29888, which is one of the most common orthopedic procedures and the second most frequent CPT code in our dataset. The TF-IDF model was able to predict CPT 29888 with AUROC of 0.99, accuracy of 1, specificity of 1 and sensitivity of 0.99. Following 5-fold cross validation, there were 203 incorrect predictions out of 44,002 operative notes. An independent examiner reviewed and relabeled the mislabeled notes, blinded to the ground truth or the predicted label. Of the 203 mislabeled notes, 198 (97.5%) notes were classified correctly by the model and had incorrect ground truth labels. Three notes were incomplete, and 2 notes were truly misclassified by model. This means that the model was able to outperform the ground truth and even identify the errors that have been made in assigning the CPT codes.

## Discussion

In this study, we systematically analyzed the ability of three commonly used NLP models to predict the 100 most common musculoskeletal CPT codes from unstructured operative notes. Our findings reject our first hypothesis as the traditional TF-IDF model with a dynamic feature space size outperformed the computationally expensive BERT model. Our results support our second hypothesis demonstrating that CPT complexity can explain up to 61% variability in AUROC. Finally, we saw that prevalence of procedures (CPT counts) only influence the model’s prediction performance in complex cases.

This study showed that NLP models can highlight human error in CPT assignment. While we did not assess the misclassification of the entire corpus, other studies, such as [5] have highlighted a manual CPT assignment error rate of 5.0%. Currently, the financial impact of billing and insurance related tasks is estimated to be around $25 billion USD [2, 32–34], and the Centers for Medicare and Medicaid Services reported that improper payments amounted to $28.91 billion USD in 2019 alone [35] highlighting the need for automated coding systems.

In this study our best performing TF-IDF model outperformed our best performing BERT model, with both models outperforming the Doc2vec models. Although this is surprising, it is similar to findings of many other studies regarding deep learning models on clinical notes. [2, 10, 17] all observed similar results in comparing performance of traditional approaches on clinical notes to deep learning models.

The superiority of traditional models to deep learning models in these tasks could be attributed to few factors. Deep learning models generally need much more data to train compared to traditional approaches, and in clinical domain, finding bigger datasets is very difficult because of privacy and security concerns and their manual labeling is very expensive. Another challenge in working with clinical notes is the presence of noise and templates. Physicians who write these notes tend to keep a template for their surgeries and only change details in the template each time. Generally, each physician has their own template which considerably affects the note representations however does not give much information as to the procedure itself, but since traditional approaches take keywords into account, they are less impacted by these templates. The same is for the noise in the data. Clinical notes are very noisy, they are usually written in a rush, hence they have a lot of misspellings, incorrect grammar, acronyms, etc. Also, in many cases these notes are forms that are saved in text format, so there are a lot of white space and non-ASCII characters included in the text. While there are different variations of BERT models, pre-trained on different datasets, all of them are clean formatted datasets which are very different from clinical notes. A possible solution to this could be to clean and preprocess the data thoroughly before feeding it to the deep learning model. We have done some preliminary experiments on selecting the relevant information from the text via traditional methods and then feeding them to deep learning models which showed promising results. However, it needs more experiments and remains as a future work.

It should also be mentioned that although BERT is an LLM, it is one of the smallest ones, and recent LLMs do not need as much data to train on downstream tasks, since they have been pre-trained on much larger datasets. Hence, BERT’s performance might not be a good representative for the other models in this group. However, considering the resources newer models require–One or more GPUs with large RAM size– it was decided not to include these models in the study design. Using models such as chatGPT [20], Llama [21], and Mistral [36] for tasks such as CPT detection, which are rather simple tasks, is quite excessive.

Another outcome of this study is that the number of notes available for a well-defined CPT in the data set, do not significantly affect the results. This information alleviates the burden of collecting large data sets for simple cohort identifications. If there are enough samples from a cohort, gathering more data is not going to improve the results significantly. It is worth noting that the least frequent CPT included in our experiments, with frequency of 127, has a AUROC value of 0.99 with TF-IDF model, hence the threshold for having enough number of samples can be quite low depending on the complexity.

This study is not without limitations. In this study, we highlighted CPT 29888 as an example of human error in the code assignment process. As our training sets were derived from data with manual CPT errors, we expect that models trained on these sets would have their own inherent flaws. Additionally, the operative reports in this study were all from the same institution. As such, it is possible that bias from overfitting may exist due to billing practices unique to our institution. Other future work includes using other types of classifiers and feature extraction methods to cover a broader range of NLP approaches for classification of clinical notes.

## Conclusion

The current study supplements the existing literature in support of using NLP to automate codifying clinical notes and to conduct quality control. Results also support use of traditional NLP approaches (i.e., TF-IDF) as proper tools for these well-defined applications. The fact that these traditional approaches are less resource intensive compared to the state-of-the-art models such as BERT, lowers the barriers to wide-spread clinical adoption.

## Supporting information

S1 Table Model performance metrics for predicting the top 100 common CPT codes from operative notes. (XLSX)

S1 Fig A visualization of the components used for calculating the complexity measure, *ratio* (solid purple circle) and *distance* (dotted circles), for CPT298888 (low complexity, seen in red), and CPT29999 (high complexity, seen in blue). Each dot represents an operative note in TF-IDF space which has been projected to 2-dimensional space using principal component analysis (PCA) [37] and TSNE [38] for visualization. The light gray notes are any procedure other than 29888 and 29999. For cleaner presentation, only a 5% subset of all operative notes is shown. For CPT 29888, the circles representing the *ratio* and *distance* fall on top of each other since all of its neighbors have the same label.
(TIF)

S2 Fig Most important keywords in identifying most (a-d) and least (e-h) common CPTs (among 100) and description for each procedure. (a) 20680: Removal of implant; deep (eg, buried wire, pin, screw, metal band, nail, rod or plate). (b) 29888: Arthroscopically aided anterior cruciate ligament repair/augmentation or reconstruction. (c) 29881: Arthroscopy, knee, surgical; with meniscectomy (medial or lateral, including any meniscal shaving). (d) 29882: Arthroscopy, knee, surgical; with meniscus repair (medial OR lateral). (e) 29450: Application of clubfoot cast with molding or manipulation, long or short leg. (f) 25607: Open treatment of distal radial extra articular fracture or epiphyseal separation, with internal fixation. (g) 27686: Lengthening or shortening of tendon, leg or ankle; multiple tendons (same incision). (h) 25390: Osteoplasty, radius OR ulna; shortening.
(TIF)
