# Supplementary figures and images for: Systematic evaluation of common natural language processing techniques to codify clinical notes
Source: PLoS One. 2024 Mar 7;19(3):e0298892. doi: 10.1371/journal.pone.0298892 (PMC10919678; doi:10.1371/journal.pone.0298892)

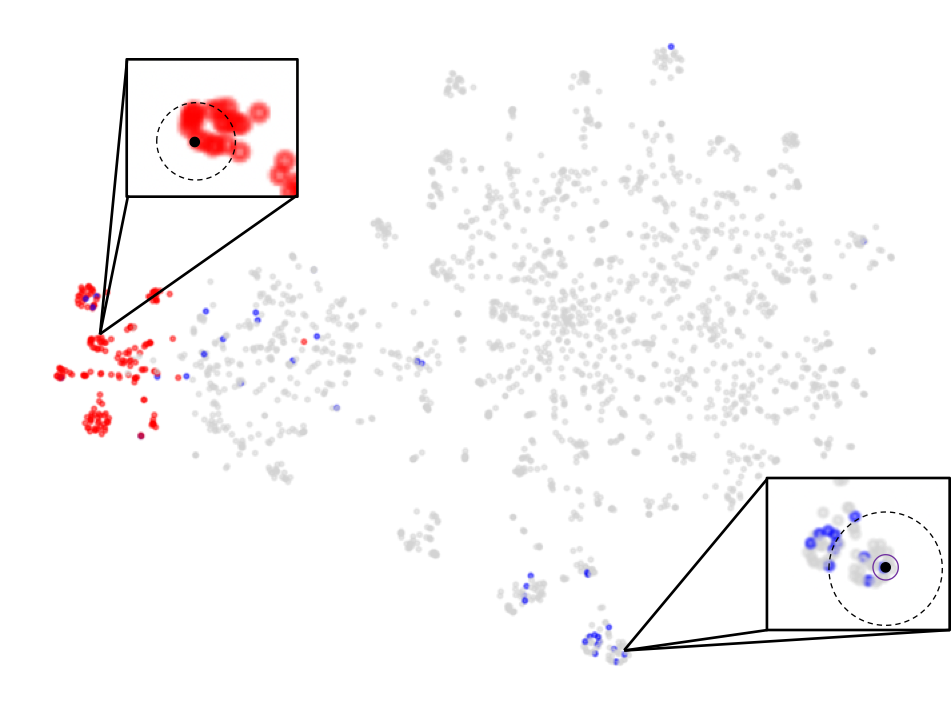

Supplement: S1 Fig — Each dot represents an operative note in TF-IDF space which has been projected to 2-dimensional space using principal component analysis (PCA) [37] and TSNE [38] for visualization. The light gray notes are any procedure other than 29888 and 29999. For cleaner presentation, only a 5% subset of all operative notes is shown. For CPT 29888, the circles representing the ratio and distance fall on top of each other since all of its neighbors have the same label. (TIF) [file pone.0298892.s002.tif]

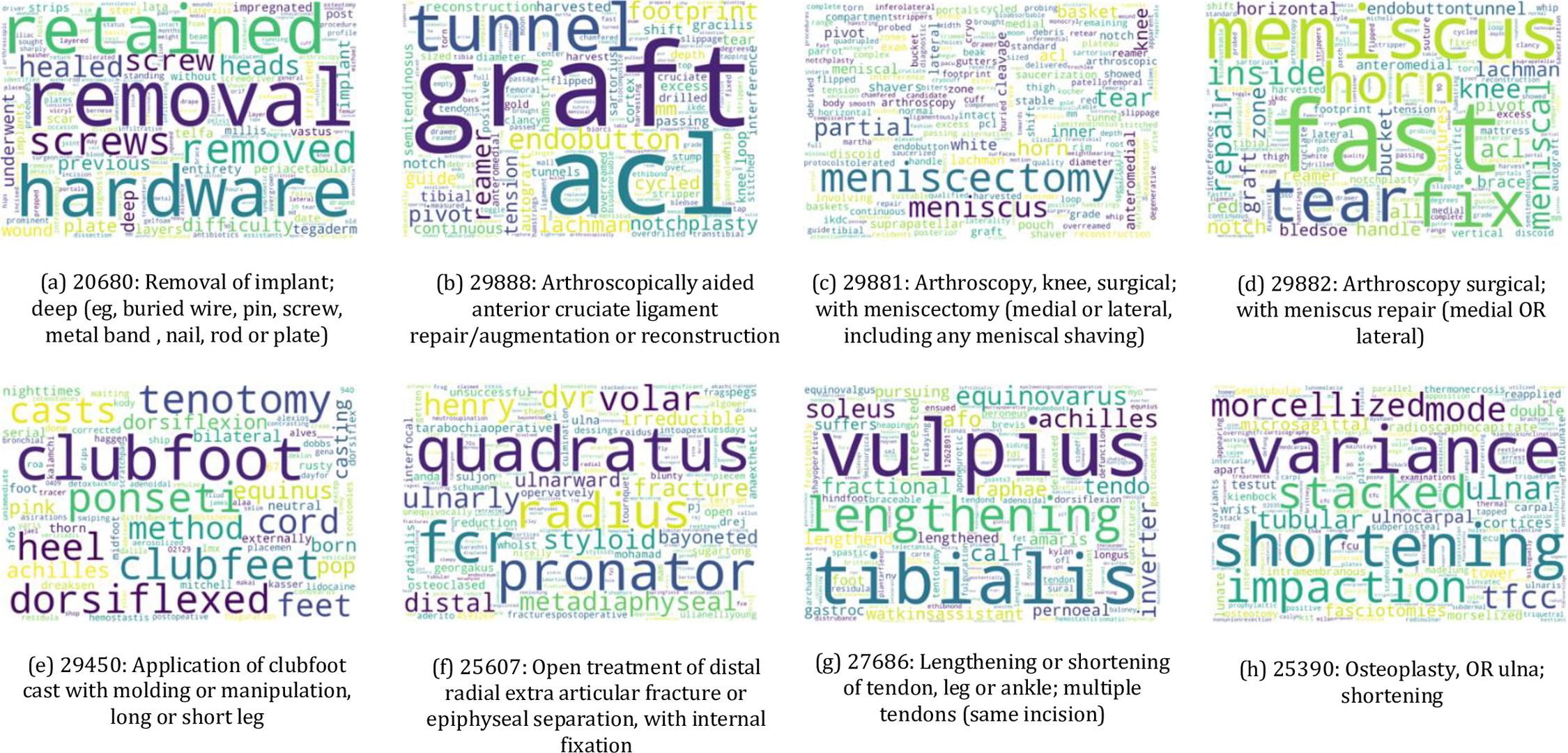

Supplement: S2 Fig — (a) 20680: Removal of implant; deep (eg, buried wire, pin, screw, metal band, nail, rod or plate). (b) 29888: Arthroscopically aided anterior cruciate ligament repair/augmentation or reconstruction. (c) 29881: Arthroscopy, knee, surgical; with meniscectomy (medial or lateral, including any meniscal shaving). (d) 29882: Arthroscopy, knee, surgical; with meniscus repair (medial OR lateral). (e) 29450: Application of clubfoot cast with molding or manipulation, long or short leg. (f) 25607: Open treatment of distal radial extra articular fracture or epiphyseal separation, with internal fixation. (g) 27686: Lengthening or shortening of tendon, leg or ankle; multiple tendons (same incision). (h) 25390: Osteoplasty, radius OR ulna; shortening. (TIF) [file pone.0298892.s003.tif]
